# Uterine Sarcomas: A Retrospective Analysis of a Cohort of 62 Patients

**DOI:** 10.7759/cureus.13349

**Published:** 2021-02-15

**Authors:** Inês Eiriz, Marina Vitorino, Fernando Gomes, Sofia Braga

**Affiliations:** 1 Medical Oncology, Hospital Prof. Doutor Fernando Fonseca, Lisbon, PRT

**Keywords:** uterine neoplasms, rare tumors, sarcomas

## Abstract

Background and objective

Uterine sarcomas are rare tumors, and they account for 4% of all uterine malignancies. These tumors are characterized by a great diversity of histological types, and current knowledge regarding their treatment is limited. The aim of our study was to analyze a cohort of patients with uterine sarcomas with respect to the histological types of their tumors, as well as their prognosis and treatment.

Materials and methods

This was a retrospective analysis involving patients diagnosed at a single center with uterine sarcoma between 2003 and 2017.

Results

The study included 62 patients; the mean age of the patients was 62 ±13 years. Carcinosarcoma was identified in 44% of cases, leiomyosarcoma in 40%, and endometrial stromal sarcoma in 13%. Endometrial stromal sarcoma was found to occur in younger women compared to carcinosarcoma (52 ±13 vs. 66 ±12 years, p=0.016); 90% of patients underwent surgery, and medical treatment was implemented in 42%. The mean overall survival (OS) was 93 ±10.65 months, and the median progression-free survival (PFS) was 12 months. There was a significant association between the stage of the disease at diagnosis and the probability of survival: mean OS was 118 months for locoregional disease vs. 44 months for metastatic disease (p<0.001). The overall five-year survival rate was 39%.

Discussion and conclusions

Uterine sarcomas are rare cancers, and they are very heterogeneous. They are also associated with a high mortality rate. Further investigational studies are required so that a more effective treatment method and individualized treatment plans can be implemented for patients with uterine sarcoma.

## Introduction

Uterine sarcomas are aggressive mesenchymal tumors, which account for 3-4% of all uterine malignant neoplasia [1]. They are a heterogeneous group of tumors that include several histologic types. The most common among them is leiomyosarcoma (around 60%) followed by endometrial stromal tumors (6-20% of uterine sarcomas) and undifferentiated sarcomas (5%) [2,3]. Adenosarcoma and carcinosarcoma constitute mixed mesenchymal and epithelial tumors. Carcinosarcoma, a malignant mixed Müllerian tumor, is a special type of uterine sarcoma since it accounts for a significant proportion of patients; however, its exact prevalence among uterine sarcomas is not well established [2]. Most uterine sarcomas occur in women over 40 years of age. Patients often present with pelvic mass or abnormal uterine bleeding; however, most patients are asymptomatic at the time of diagnosis [3,4].

Leiomyosarcoma arises from smooth muscle of the uterine wall and frequently presents with diseases limited to the uterus. Cure rates range from 20 to 60%. However, relapse rates are approximately 70% for the early stages. The site of metastasis is often the lung or liver [3,5]. Endometrial stromal tumors typically occur in younger women [4]. It has an indolent behavior, and local recurrences and distant metastasis can occur even 20 years after the diagnosis [5]. Undifferentiated sarcomas usually result in a poor prognosis because they grow quickly [5]. Carcinosarcomas are dedifferentiated (metaplastic) carcinomas containing carcinomatous and sarcomatous elements developing from a single malignant epithelial cell clone. Usually, these occur in older women and the prognosis is very poor [6].

Classically, these various types of tumors have been treated as a single disease, with rather disappointing results in the advanced setting. Survival generally depends on the stage, with a five-year survival of 65% for localized disease and less than 30% for stage IV disease [7].

In this study, we aimed to review our experience with uterine sarcomas, in order to analyze their clinical and histopathological features, to discuss therapeutic difficulties associated with these tumors, and compare our findings with previously published data.

## Materials and methods

This was a retrospective observational single-center analysis conducted at a Portuguese Oncology Department. This study was approved by the local health ethics committee (number 43/2019). Clinical data were obtained for 62 patients who were more than 18 years old and diagnosed with uterine sarcoma from January 2003 to December 2017. Patients who did not have definitive histologic diagnosis were excluded. Epidemiological and clinical data collected from patients’ medical records were related to the following parameters: age at diagnosis, histologic types, and clinical and pathologic staging according to the International Federation of Gynecology and Obstetrics (FIGO). Types of treatments received were also reviewed [surgery, chemotherapy (CT), and radiotherapy (RT)], as well as prognostic variables such as date of first relapse or progression of the disease, site of metastasis, and date of death or last time seen.

Statistical analysis was performed using IBM® SPSS® Statistics software version 24 (IBM, Armonk, NY). Categorical data were presented as counts and percentages and were analyzed with the chi-square test and Fisher’s exact test, as appropriate. The skewed distributions were described with medians and interquartile ranges (IQR). Normal distributions were described with means and standard deviations and were compared by using the Student’s t-test. Differences between more than two groups were evaluated by using one-way analysis of variance (ANOVA), followed by the Tukey-Kramer test when findings with the ANOVA model were significant. Survival analysis was performed using the Kaplan-Meier method, and the log-rank test was used to assess differences among survival rates. The outcomes of interest were overall survival (OS) and progression-free survival (PFS). For survival analysis, we grouped FIGO stages I to III into local or locoregional disease and stage IV into metastatic disease due to the small size of the sample. To evaluate the outcomes of CT and RT, we grouped stages I and II into early disease and stages III and IV into advanced disease, because of recommendation similarities. Survival analysis by histologic type was performed after the exclusion of adenosarcoma and undifferentiated sarcoma due to the small sample (two patients). All p-values were two-sided and a p-value of <0.05 was considered statistically significant.

## Results

Out of an initial number of 72 patients, we identified 62 patients who were diagnosed with a specific histologic subtype of uterine sarcoma. The demographic and clinicopathologic features of the entire cohort are presented in Table 1. The mean age was 62 ±13 years; 27 patients (43.5%) had carcinosarcomas, 25 (40.3%) had leiomyosarcomas, eight (12.9%) had endometrial stromal sarcoma, and there was one case each with adenosarcoma and undifferentiated sarcoma. The distribution of patients according to FIGO staging at diagnosis was as follows: 25 patients (40.3%) in stage I, 10 patients (16.1%) in stage II, seven patients (11.3%) in stage III, and 20 patients (32.3%) in stage IV. Fifty-six (90.3%) cases underwent surgery and 29 (46.8%) had RT. Regarding medical treatment, 21 (33.9%) patients were treated with CT (doxorubicin and ifosfamide as the main protocol in 38% of cases), three (4.8%) with hormonal therapy, and two (3.2%) with tyrosine-kinase inhibitors. Twenty-five (40.3%) women experienced relapse or progression of the disease. The main site of metastasis was lung with 23 events (46.9%), followed by peritoneum and liver with 10 (20.4%) and seven cases (14.3%) respectively. Bone, lymph nodes, and vagina were less common locations for metastasis, accounting for four, three, and two cases respectively.

**Table 1:** Characteristics of the study population FIGO: International Federation of Gynecology and Obstetrics

Variables	N	%
Total population	62	100
Age (years), mean	62	
Histology		
Leiomyosarcoma	25	40.3
Carcinosarcoma	27	43.5
Endometrial stromal sarcoma	8	12.9
Undifferentiated sarcoma	1	1.6
Adenosarcoma	1	1.6
FIGO staging		
I	25	40.3
II	10	16.1
III	7	11.3
IV	20	32.3
Surgery	56	90.3
Chemotherapy	21	33.9
Hormone therapy	3	4.8
Tyrosine kinase inhibitors	2	3.2
Radiotherapy	29	46.8
Relapse or progressive disease	25	40.3
Metastasis sites		
Lung	23	46.9
Peritoneum	10	20.4
Liver	7	14.3
Vagina	2	2
Lymph nodes	3	6.1
Bone	4	8.1
Death	32	51.6

The mean OS was 93 ±10.65 months, and the median PFS was 12 months [95% confidence interval (CI): 8.3-15.7]. We found a significant association between the stage of disease at diagnosis and probability of survival: local or locoregional disease had a mean OS of 118 months vs. 44 months for metastatic disease (log-rank p<0.001). According to FIGO staging, the mean OS for stage I was 119 months, that of stage II was 91 months, stage III was 76 months, and stage IV was 45 months (log-rank p=0.001). The median PFS according to FIGO staging was not statistically significant: 23 months for stage I, 13 months for stage II, and nine months for stages III or IV (log-rank p=0.196).

**Figure 1:**
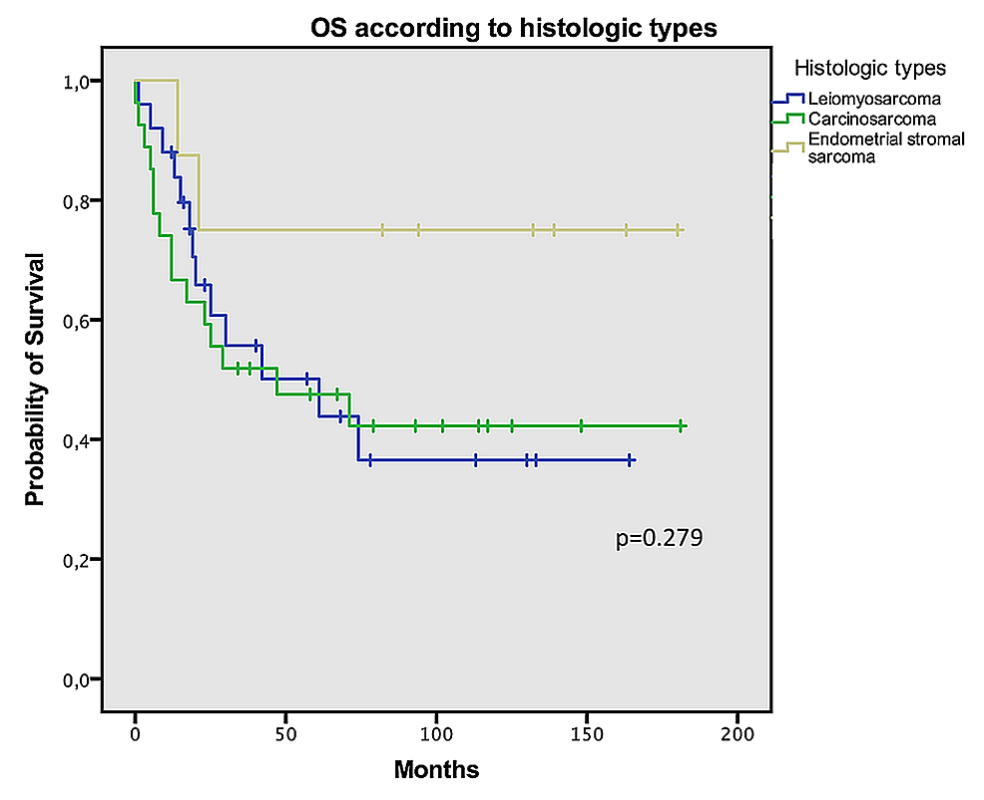
Overall survival according to histology OS: overall survival

With regard to the three most prevalent histologic types of uterine sarcomas in our cohort, we also found differences, although not significant, in survival depending on pathologic features. Endometrial stromal sarcoma had a better survival (139 months), compared with leiomyosarcoma (61 months) and with carcinosarcoma, which had the worst prognosis (47 months, log-rank p=0.279) (Figure 1). Median PFS was also better in patients with endometrial stromal sarcoma (23 months) than in those with leiomyosarcoma (10 months) or carcinosarcoma (12 months, log-rank p=0.622). 

Patients with endometrial stromal sarcoma were significantly younger than patients with carcinosarcoma (51.5 ±13.5 vs. 65.7 ±12 years, p=0.016). There was no statistical difference between the mean age of leiomyosarcoma patients and that of patients with other histologic types.

**Figure 2:**
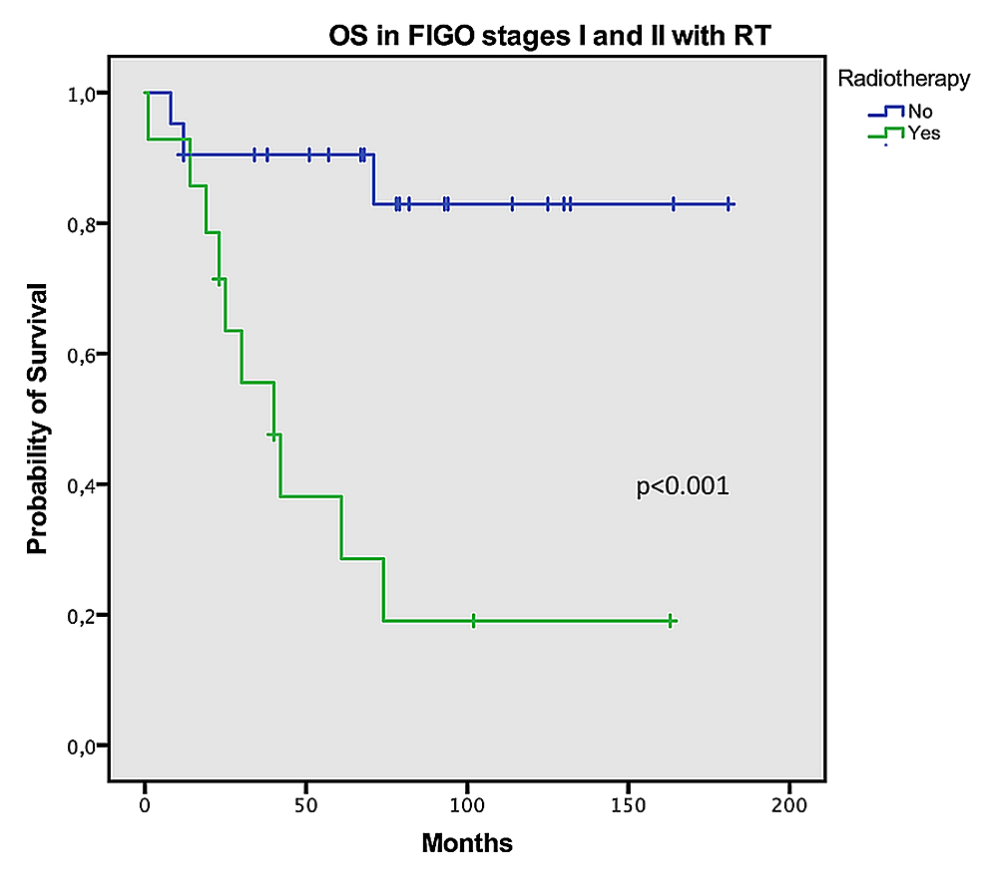
Overall survival in FIGO stages I and II with radiotherapy OS: overall survival; FIGO: International Federation of Gynecology and Obstetrics; RT: radiotherapy

**Figure 3:**
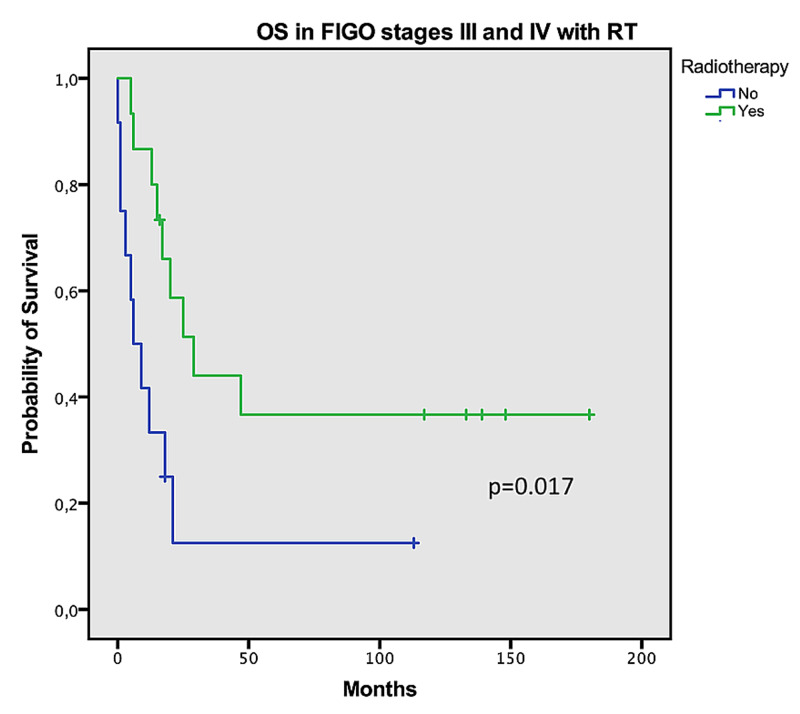
Overall survival in FIGO stages III and IV with radiotherapy OS: overall survival; FIGO: International Federation of Gynecology and Obstetrics; RT: radiotherapy

In patients with uterine sarcomas with FIGO stages III or IV who underwent CT, the mean OS was found to be better compared to those who did not go undergo CT; however, this difference was not statistically significant (55 vs. 49 months, log-rank p=0.741). In this group of patients, the median PFS was greater by five months in those treated with CT (nine vs. four months, p=0.81). In early disease (stages I and II), OS was more prolonged in patients who were not treated with adjuvant RT (156 vs. 60 months, log-rank p<0.001) (Figure 2). In advanced disease (stages III and IV), undergoing RT was associated with increased median OS in these patients (79 vs. 21 months, log-rank p=0.017) (Figure 3).

At the time of analysis, 51.6% of the population was already dead. The survival rate at five years was 38.7%.

## Discussion

Uterine sarcomas are rare malignant tumors with a generally poor prognosis. The heterogeneity of histologic types and biologic behavior is considerable among these patients. Owing to its rarity, there is scarce data concerning this type of cancer, and most studies on it have been retrospective. Therefore, we could draw some important conclusions from our cohort of 62 patients diagnosed in a period of 15 years. In the present study, the mean OS of the entire population was 93 months, and the five-year survival rate was 38.7%, which seems better compared to some reports in the literature [8,9]. Some authors have reported an estimated five-year survival rate of 36% in surgically treated patients with stage I uterine sarcoma [8]. One study has documented a five-year survival rate of 33% for 23 patients with early-stage uterine sarcoma, most of whom were treated with a combination of surgery and pelvic irradiation [9]. The surgical expertise of the medical team influences survival and the majority of uterine sarcomas have no established diagnosis of malignancy before oncologic surgery [5]. An oncological surgery with staging by an experienced team is the most important step in the treatment of these patients [10].

Our results have demonstrated that the stage of the disease is significantly associated with survival, and histopathological type also has an impact on survival. Patients with endometrial stromal tumors tend to be younger [5], have a good prognosis, and can be cured by oncological surgery [11]. Low-grade endometrial stromal sarcomas generally affect women between the ages of 40 and 55 years, and extrauterine pelvic extension is found in one-third of such cases. Endometrial stromal sarcomas are indolent tumors, but late recurrences may occur in the pelvis and abdomen. Similar to our cohort, other studies have found that patients with endometrial stromal sarcomas have a more favorable prognosis than patients with other histological types (p<0.001). Moreover, these studies have also reported the importance of stage (p<0.001) and age (p<0.001) as prognostic predictors [12-14]. In fact, in our cohort, patients in earlier stages had a much better prognosis (mean OS for stage I: 119 months), and this is in line with previous studies [15]. In our population, age had no association with OS, but younger patients had better PFS than the older ones (63 months of PFS in patients aged below 40 years vs. one month in women over 75 years, log-rank p=0.011).

Due to a lack of large randomized controlled trials, most treatment guidelines are based on small and retrospective studies in the literature. There are little published data about these tumors since these are rare and heterogeneous, and the patients are usually elderly and symptomatic, a difficult population for inclusion in clinical trials. The mainstay of treatment for early-stage uterine sarcomas is surgery, and adjuvant RT is controversial [16]. In a large retrospective cohort of 3,650 patients from a National Oncology US Database, postoperative RT significantly improved local control of the disease, with a 53% reduction in the risk of locoregional failure at five years compared to surgery alone. However, it did not significantly improve OS [17]. A phase III randomized trial has assessed the efficacy of adjuvant pelvic RT in uterine sarcoma where patients with stage I and II were randomized to adjuvant RT to the pelvis or observation after total abdominal hysterectomy and bilateral salpingo-oophorectomy [10]. With a six-year median follow-up, adjuvant RT resulted in significant local control in all patients (22 vs. 40%, p=0.004) [10]. However, RT had no impact on OS and PFS, and the difference in local disease control was significant for carcinosarcoma, but not for leiomyosarcoma [10]. In our cohort, RT did not improve OS in early stages (I and II), but in advanced disease (stages III and IV), performing RT was associated with improved median OS with statistical significance. Patients with stage IV disease who had undergone surgical resection were also treated with postsurgical RT. We believe that the difference found in survival between early and advanced stages might be due to the fact that more patients received RT in advanced stages, which might have improved disease control.

Regarding adjuvant CT, there is little evidence in the literature supporting its use except for carcinosarcoma. In a retrospective analysis, no significant impact of CT on survival was observed, which was slightly inferior at three years. The authors postulated that this was mainly due to the fact that patients who were eligible for CT were in the higher stages of the disease [10]. These patients are frequently elderly, and hence CT could be very toxic; it is important to emphasize that the mean age of our patients was 62 years, which is a very old population and, therefore, frailer.

In our study, in patients with uterine sarcomas in FIGO stages III or IV who underwent CT, the median OS and median PFS were better compared to those who did not undergo CT, albeit without statistical significance. This supports the previously stated hypothesis that the impact of CT is low because patients eligible for this modality are usually in the late stage of the disease and have a poor prognosis, regardless of treatment.

It is important to assess hormonal receptors in these tumors; in our cohort, we could not reach a conclusion regarding the benefit of hormonal therapy due to the small number of patients treated.

Our study has some limitations. Primarily, it involved a small observational cohort. Moreover, due to its retrospective nature, few conclusions could be reached concerning the treatments performed. Nevertheless, we were able to elicit some important prognostic information.

## Conclusions

Uterine sarcomas are rare but still associated with significant mortality. Although limited by the small sample size and retrospective nature, our study revealed certain relevant findings. The prognosis of uterine sarcomas seems to be dependent on histological subtype and disease stage. Endometrial stromal sarcoma and early-stage disease are associated with better outcomes. Moreover, endometrial stromal sarcoma patients were younger in age. Therefore, biological behavior might be significantly different between histologic types. A differentiation between these types should be made in order to improve the management of these patients in future studies. Another important technique is molecular analysis, which, in soft tissue sarcomas, has enabled advances in treatment approach and, consequently, in survival outcomes.

## References

[1] D'Angelo E, Prat J (2010). Uterine sarcomas: a review. Gynecol Oncol.

[2] Amant F, Moerman P, Neven P, Timmerman D, Van Limbergen E, Vergote I (2005). Endometrial cancer. Lancet.

[3] Ramondetta LM, Bodurka DC, Deavers MT, Jhingran A (2006). Uterine sarcomas. MD Anderson Cancer Care Series: Gynecologic Cancer.

[4] (2003). Pathology and Genetics of Tumours of the Breast and Female Genital Organs: WHO Classification of Tumours, 3rd Edition, Volume 4. Lyon, France. IARC Press.

[5] Gadducci A, Cosio S, Romanini A, Genazzani AR (2008). The management of patients with uterine sarcoma: a debated clinical challenge. Crit Rev Oncol Hematol.

[6] Matsuo K, Takazawa Y, Ross MS (2018). Proposal for risk-based categorization of uterine carcinosarcoma. Ann Surg Oncol.

[7] Kapp DS, Shin JY, Chan JK (2008). Prognostic factors and survival in 1396 patients with uterine leiomyosarcomas: emphasis on impact of lymphadenectomy and oophorectomy. Cancer.

[8] Piver MS, Lele SB, Marchetti DL, Emrich LJ (1988). Effect of adjuvant chemotherapy on time to recurrence and survival of stage I uterine sarcomas. J Surg Oncol.

[9] Gadducci A, Fabrini MG, Facchini V (1989). Surgery and radiotherapy in the treatment of early stage uterine sarcomas. Eur J Gynaecol Oncol.

[10] Reed NS, Mangioni C, Malmström H (2008). Phase III randomised study to evaluate the role of adjuvant pelvic radiotherapy in the treatment of uterine sarcomas stages I and II: a European Organisation for Research and Treatment of Cancer Gynaecological Cancer Group Study (protocol 55874). Eur J Cancer.

[11] Dionigi A, Oliva E, Clement PB, Young RH (2002). Endometrial stromal nodules and endometrial stromal tumors with limited infiltration: a clinicopathologic study of 50 cases. Am J Surg Pathol.

[12] Nordal RR, Kristensen GB, Stenwig AE, Nesland JM, Pettersen EO, Trope CG (1997). An evaluation of prognostic factors in uterine carcinosarcoma. Gynecol Oncol.

[13] Kelly KL, Craighead PS (2005). Characteristics and management of uterine sarcoma patients treated at the Tom Baker Cancer Centre. Int J Gynecol Cancer.

[14] Livi L, Andreopoulou E, Shah N, Paiar F, Blake P, Judson I, Harmer C (2004). Treatment of uterine sarcoma at the Royal Marsden Hospital from 1974 to 1998. Clin Oncol (R Coll Radiol).

[15] Chang KL, Crabtree GS, Lim-Tan SK, Kempson RL, Hendrickson MR (1990). Primary uterine endometrial stromal neoplasms. A clinicopathologic study of 117 cases. Am J Surg Pathol.

[16] García-Martínez E, Egea Prefasi L, García-Donas J, Escolar-Pérez PP, Pastor F, González-Martín A (2011). Current management of uterine sarcomas. Clin Transl Oncol.

[17] Sampath S, Schultheiss TE, Ryu JK, Wong JY (2010). The role of adjuvant radiation in uterine sarcomas. Int J Radiat Oncol Biol Phys.

